# Health human resources planning in Canada—Part I: Opportunities and challenges for pharmacy

**DOI:** 10.1177/17151635231201802

**Published:** 2023-09-28

**Authors:** Zubin Austin, Natalie Crown

**Affiliations:** Leslie Dan Faculty of Pharmacy, University of Toronto, Toronto, Ontario; Institute for Health Policy, Management and Evaluation – Temerty Faculty of Medicine, University of Toronto, Toronto, Ontario; Leslie Dan Faculty of Pharmacy, University of Toronto, Toronto, Ontario

**Knowledge into Practice**

- Pan-Canadian health human resources (HHR) planning is required to ensure a sustainable, equitable and cost-effective health care system.
- Pharmacy has traditionally not been part of Health Canada’s HHR planning processes, despite being the third largest health profession.
- As the scope of practice expands and reliance on pharmacists in primary care grows, there is greater interest in including pharmacy in pan-Canadian HHR planning.
- Pan-Canadian HHR planning in pharmacy is complicated by different provincial scopes of practice, the complex interplay of for-profit/private-sector employers and the rapid pace of practice change.
- Aligning pharmacy interests and data sets to conform with HHR and Health Canada planning processes will enhance pharmacy’s ability to manage its workforce needs in the future.

## Background

This is the first in a series of articles written to help the pharmacy professions understand the evolution of and processes used in health human resource (HHR) planning in Canada. Understanding how HHR planning occurs can help the profession, employers, professional associations, educators and individual pharmacists/regulated technicians align interests and efforts in a more productive manner to support systemwide efforts to ensure a sustainable, equitable and cost-effective health care system for all Canadians, now and in the future.

It is generally accepted that there is a crisis in the Canadian health care system.^1^ Reports of large numbers of Canadians unable to access primary care services,^2^ or practitioners’ experiences of occupational stress, burnout and mental health challenges arising from under-resourced workplaces,^3^ or anecdotal reports of well-qualified internationally educated health professionals driving taxi cabs or working in factories^4^ all highlight gaps and failures in planning, organization, management and administration of Canada’s health care systems. The COVID-19 pandemic stressed health care systems as never before^5^: the fortitude of the health workforce in rising to pandemic-related challenges was truly remarkable and speaks to the motivation and dedication of hundreds of thousands of individuals who work as regulated health professionals and nonregulated health workers. With the declaration by the World Health Organization (WHO) of the end to COVID-19 as a public health emergency, the impacts on health care systems—and the health workforce—in Canada have become more evident.^6^

A strong, sustainable, responsive, evidence-based health care system is a cornerstone of Canadian society and life^7^ and, until relatively recently, something most Canadians simply took for granted. The Canada Health Act is Canada’s federal legislation that lays out both the primary objectives of health policy as well as enables a national system of publicly funded health care insurance. At its core, the CHA focuses on “facilitating reasonable access to health services without financial or other barriers” on a prepaid basis, preventing direct charges at the point of care delivery.^8^

Health care delivery is complex and expensive and consists of multiple interconnected components. At its most basic, health care systems require people, places for these people to work and products for these people to use in delivering service and care. In addition, there are administrative and other support structures that surround the people, places and products to ensure health care work is safe, effective and cost-efficient. While places (such as hospitals) or products (such as biologics or other expensive medications) are certainly important and garner great public attention, people (i.e., the health care workforce) are by far the largest, most costly and most important component of any health system.^9^ It is also these people who define the actual quality and success of health care systems: without a motivated, engaged, qualified and resilient workforce, it simply does not matter how many hospitals get built or what medications are listed on provincial formularies.

Health Canada has identified a Canadian HHR crisis that has been further worsened by the COVID-19 pandemic and subsequent political, economic and societal fractures.^10^ As a result, there has been significant political interest in HHR planning as a tool to prevent further deterioration in accessibility to health care services, enhance safety, quality and cost-effectiveness and to ensure sustainability of our system.

It is tempting to believe that HHR is a government responsibility or something that is too large for ordinary citizens or average pharmacists to worry about, contribute to, participate in or engage with in any meaningful way. While the outcomes of HHR planning certainly do affect the day-to-day lives of ordinary citizens and average pharmacists alike, it is essential to recognize that HHR planning needs to engage all Canadians in some way to be meaningful. Specifically, if the pharmacy profession and individual pharmacists are not actively engaged in HHR planning processes, the needs and contributions of the profession will be misunderstood, overlooked and underestimated. Understanding the objectives of HHR planning, the processes that are used, the strengths and limitations of methodologies relied upon and the ways in which individual citizens and practitioners can contribute to and influence decision-making is not only important for engaging all Canadians in this work but also for generating the most accurate data sets upon which major planning and resource allocation decisions are made.

## What is HHR planning?

HHR planning is generally described as the process of identifying the right number of health care workers with the right knowledge, skills, attitudes and qualifications performing the right tasks in the right places at the right time to achieve the right predefined health targets for a population.^11^ Simply, effective health workforce planning balances current and future societal health needs with workforce capacity and resource allocation. Effective health workforce planning prevents the “yo-yo” that often plagues professions, including pharmacy, whereby an actual or perceived practitioner shortfall triggers reactive responses to immediate staffing issues and resulting oversupply. For HHR planning to succeed, it must be conducted on a backbone of accurate, timely, accessible and actionable workforce data obtained through the engagement of diverse partners.

**Mise En Pratique Des Connaissances**

- La planification pancanadienne des ressources humaines en santé (RHS) est nécessaire pour garantir un système de soins de santé durable, équitable et rentable.
- Traditionnellement, la pharmacie ne fait pas partie des processus de planification de Santé Canada en matière de RHS, bien qu’elle soit le troisième domaine en importance dans le secteur de la santé.
- À mesure que le champ d’exercice s’élargit et que les pharmaciens sont de plus en plus sollicités pour les soins primaires, l’intérêt d’inclure la pharmacie dans la planification pancanadienne des RHS devient plus marqué.
- La planification pancanadienne des RHS en pharmacie est compliquée par les différents champs d’application provinciaux, l’interaction complexe entre les employeurs du secteur privé et à but lucratif et le rythme rapide de l’évolution de la pratique.
- L’harmonisation des intérêts et des bases de données de la pharmacie avec les processus de planification des RHS et de Santé Canada améliorera la capacité des pharmacies à gérer leurs besoins en main-d’œuvre à l’avenir.

The central challenge for national HHR planning in pharmacy involves establishing consensus on critically important issues (such as scope of practice) for which provincial variability exists. An HHR planning system that is overly focused on local/provincial needs will lead to inconsistent/asymmetric care and service delivery nationally, limits labour mobility and reduces workforce flexibility/responsiveness across the country.

Pan-Canadian HHR planning is sometimes criticized as being impersonal, bureaucratic and top-down in its orientation, conveying a sense that it excludes or ignores individuals’ stories and local needs in favour of mathematical formulae. HHR planners try to address these concerns by developing frameworks to reduce the complexity of their work and make it more accessible to individual citizens, patients, practitioners and professions to contribute meaningfully to data collection and analysis. Currently, the Health Canada framework for HHR planning has identified 5 core data-driven areas for focus.^12^ The intersection of these 5 distinct but related areas forms the foundation of a pan-Canadian vision of HHR as well as the policy platform upon which pan-Canadian solutions to the current HHR crisis will be built. It is essential for the pharmacy profession, and other individual professionals, to understand how this process works and the influence it will have on the workforce in the years ahead.

### Recruitment for future needs

The Canadian health care system will experience significant increasing demand for care, especially in the community-based and ambulatory care settings. As a result, there will be a need for a more flexible and mobile pan-Canadian professional labour force able to move seamlessly across provinces and local health systems as the need for their services rises in some locations and falls in others.^13^ The WHO projects that by 2030, there will be a shortage of more than 15 million health care workers globally.^14^ This poses a particular risk for the Canadian health care system (and pharmacy in particular), which has an unusually high reliance on internationally educated health professionals compared with other countries, due to chronic underproduction of domestically educated health professionals.

Canada’s population continues to grow at a record-setting pace. Canada’s population has reached a new milestone: 40 million people. In 2022, the number of Canadians grew by more than 1 million people in a single year, the highest annual population growth since the postwar baby boom in 1957. Notably, international migration accounted for almost all (96%) of the growth recorded in 2022. Some projections suggest that by 2036, immigrants and second-generation individuals together could represent nearly 1 in every 2 Canadians. Recruiting health care professionals who reflect the diversity of Canada’s population is essential to ensuring culturally safe and inclusive health care that achieves optimal health outcomes.

### Retention of health care workers

Postpandemic, there have been significant concerns raised regarding the exodus of experienced professionals (particularly nurses, physicians and personal support workers [PSWs]) from health care work. In part, this reflects workforce demographics, as baby boomers reach and exceed normal retirement age. It also reflects the reality that from 2020 to 2023 during the peak pandemic, many health workers stayed past their normal retirement age to serve and continue to provide care; as the pandemic crisis has abated, these individuals are retiring in large numbers. Most likely, however, is the reality that health care work is extremely draining and difficult, that salaries have not kept pace with demands and that workplace conditions (e.g., workload, increasing responsibilities, decreased supports) have deteriorated dramatically during and after the pandemic. Some professions have HHR planning data to describe the problems: for example, the Registered Nurses Association of Ontario has identified that 15.6% of nurses have left or are very likely to leave the nursing profession for a new occupation postpandemic.^15^ Fifty percent of PSWs have left the health care sector after less than 5 years in the field.^16^

Importantly, there are weak or nonexistent comparable data in the pharmacy professions to understand retention issues related to pharmacists and regulated pharmacy technicians in community and hospital practice. The little data we do have cast a grim picture. CPhA has published some of the findings of the 2023 Canadian Pharmacy Mental Health and Workforce Wellness Survey.^17^ In that survey of more than 1000 pharmacy professionals, only 4 of 10 pharmacists reported feeling fulfilled by their work. Furthermore, 43% reported they were considering reducing their hours, and 28% reported they were likely or somewhat likely to leave the pharmacy sector within the next year.

### Workforce mental health and well-being

The toll of the pandemic on health workforce well-being has been significant; according to the Ontario Science Advisory Table, severe emotional, physical and mental exhaustion among Canadian health care workers (including pharmacists) increased to 60% by mid-2021, more than doubling from early 2020 at the start of the pandemic.^18^ What has happened since mid-2021 is currently being studied. In the CPhA survey,^17^ only 1 in 3 pharmacy professionals rated their mental health as good or very good in the previous year. Workforce well-being connects directly to issues of workforce retention but also to quality and safety of care provided. Facile politically driven solutions to expedite licensure of newly graduated practitioners or international graduates to address shortages will not fix the HHR crisis if these newly licensed individuals are thrust into the same workplaces where mental health and well-being are currently being severely compromised. Identifying causes and implementing interventions to address underlying reasons for mental health and well-being problems is necessary to ensure another generation of workers does not burn out and perpetuate current staffing and HHR shortages.

### Data to support effective workforce planning and management

The size, diversity and responsibilities of the health workforce can sometimes defy comprehension or explanation. Despite (or perhaps because) of this complexity, the need for careful planning and management is essential to prevent waste, ensure appropriate backup and redundancies, establish proportionate checks and balances within the system and ultimately ensure optimal utilization of health care resources and funding. In other countries—including Australia, the United Kingdom and the United States—there is a national, data-driven approach to planning and management. The Organization for Economic Development and Cooperation (OECD) has noted that compared with these other comparator jurisdictions, Canada lags in terms of the robustness of national workforce data that can be used for planning and management.^19^ These data range from simple demographics (e.g., age and ethnocultural diversity) to practice-specific issues (e.g., specializations) to workplace characteristics (e.g., workloads). Within Canada, some professions (notably nursing) have better established infrastructure across all arms of the profession to capture and analyze these data. Despite being the third-largest health professional group in Canada, the pharmacy professions (including regulated pharmacy technicians) lack this kind of data infrastructure to inform evidence-based decision- and policy-making.^20^ Currently, within pharmacy, there is an uncoordinated patchwork of data sets: for example, large corporate employers maintain their own human resource records and generally do not share or make this available widely for competitive reasons, while independent pharmacies rarely even collect (and so cannot report) such data. Hospital pharmacy data are disaggregated from the rest of the profession, while data on “technicians” can be clouded by the continuing evolution of the regulated pharmacy technician and the nonregulated pharmacy assistant roles.

### Productivity and models of care

The OECD estimates that in professions for which reasonable or robust data exist, there are significant misalignments between tasks completed and qualifications of personnel completing these tasks, leading to economic inefficiencies and decreased health care productivity.^21^ For example, it is estimated that close to 80% of nurses and 76% of physicians perform routine daily tasks for which they are overqualified.^21^ This means they are not available to perform other, more meaningful and qualification-appropriate tasks, which leads to the perception of “skills shortages.”^21^ Within pharmacy, only limited empirical data have been generated, and instead there are only anecdotal reports that pharmacists are “overqualified” for the work they perform. Expansions in scope of practice are a source of pride for many pharmacists and our professional advocacy bodies. Indeed, across Canada, recent expansion of scope of practice in BC and Ontario to include activities such as common ailments prescribing has taken small steps toward the substantive work that remains to harmonize full scope of practice for all pharmacists across Canada. This highlights the way in which the transfer of tasks from physicians to pharmacists affects HHR planning and the workforce. Regulatory change to enable scope-of-practice expansion has affected virtually every practising pharmacist in some way and is the result of extraordinary political pressure focused on the HHR crisis and a perception that primary care access and quality are chaotic. There is strong political motivation for scope of practice expansion for pharmacists to be the “answer” to problems plaguing health care delivery today, yet the decisions to enable it were made with limited evidence regarding the readiness of the pharmacy workforces to implement it in a sustainable, high-quality, effective and efficient manner. This raises legitimate concerns about how successful such high-profile, sweeping changes will be and what the impact might be on an already overworked, overstressed and increasingly fragile pharmacy workforce.

## HHR planning as an opportunity for action

Focusing attention on the 5 core areas highlighted above has helped HHR planners start to prioritize innovations and opportunities to use a more systematic, methodical and data-driven approach to decision- and policy-making. HHR relies on robust data that can be analyzed in many ways and leveraged to help professionals, planners, politicians and the public understand implications of certain policy choices over other ones. Without HHR data, planning truly becomes guesswork: while there will always be uncertainty, ambiguity and the risk of overconfident decision-making with HHR planning, data provide at least some firmer foundation for informed analysis.

Currently, Health Canada has adopted an HHR planning model that emphasizes the principle of “act locally, think nationally.”^12^ In large part, this reflects the somewhat disjointed nature of Canada’s federation, in which federal funding, provincial authority and municipal/regional delivery all compete for attention and priority. Long-standing legislation and precedence mean that certain aspects of HHR planning (for example, provincial regulation of professions rather than a single national regulatory body) are unlikely to shift dramatically. Incremental improvements that work within difficult-to-change legal and historical structures will be prioritized. In considering the 5 core areas of HHR planning noted above, several important themes are emerging:

1. *Recruitment for future needs*: Health Canada is working to create structures and processes to reimagine approaches to recruitment. Several areas of importance for the pharmacy profession are starting to emerge, including the unusually high reliance on international pharmacy graduates across Canada, the “stickiness” of interprovincial movement by pharmacists and the relatively major differences in scopes of practice for pharmacists from province to province. HHR planning in pharmacy will need to focus on the number and distribution of pharmacy degree programs across Canada: with a population of 40 million people, there are currently only 12 pharmacy schools across the country, and the number of graduates produced each year does not come close to the demand from pharmacy employers. While pharmacy has a uniform pan-Canadian accreditation framework for pharmacy degree programs, variation in provincial scopes of practice introduce challenges for interprovincial migration of the pharmacy workforce, limiting the ability of individuals to move from low-demand jurisdictions to high-demand jurisdictions. While recent unilateral government involvement in regulatory bodies’ policy-making has resulted in changes, this kind of intervention is generally unhelpful for HHR planning as it is driven by political expediency, not data-informed analysis. There are even less data available regarding the pharmacy technician workforce and even less interprovincial mobility of this large and growingly important part of the pharmacy workforce.
2. *Retention of health care workers:* Career pathways within professions has been identified as an important tool for keeping the health workforce engaged over a 30-, 40- or even 50-year career life span. Such pathways require data to understand needs, career trajectories and opportunities that are available both within specific organizations and across the profession and provinces. Some professions (e.g., nursing) have a very well-developed career ladder and a highly differentiated workforce ranging from licensed practical nurses to nurse practitioners, each with their own level of education, skills qualification, responsibility and career opportunities, or the medical profession with its very clearly demarcated system of specialties and Royal Colleges. Currently, pharmacy issues a single license that covers practice in hospital, community, primary care, ambulatory care, industry and every other practice setting. There is no currently available data that can help the profession visualize the career tracks and trajectories of individuals within the profession and the ways in which these encourage retention while respecting work-life flexibility and individual’s aspirations. While some formal structures (e.g., hospital pharmacy residency programs) exist that differentiate career trajectories, they are limited and within that sector of hospital pharmacy do not necessarily support career laddering. An important lesson from the nursing and medical professions has been the value of clarification of career pathways within professions as a tool to enhance retention over a lifetime of work, and this is a potential future direction for pharmacy HHR planning.
3. *Workforce mental health and well-being:* Arguably the single most urgent HHR planning priority (in pharmacy and system-wide) is to address the corrosive burnout that drives people to leave their professions and that results in underperformance, quality-related issues and heightened rates of error.^13^ Since most health professions are overwhelmingly female-dominated and most (including pharmacy and nursing) are increasingly composed of racialized and other equity-deserving groups, these well-being issues also have an equity and inclusivity dimension. For example, there is limited research in nursing (and even less in pharmacy) detailing the workplace abuse, bullying and violence experienced on a regular basis that has profound implications for staffing, retention, practice-based performance and morale.
4. *Data to support effective workforce planning and management:* A current priority in HHR is to coordinate planning and signpost accountability for the development of “minimum data standards.” These standards are essential to facilitate jurisdictional and cross-professional comparisons and to support systemwide and nationwide planning rather than profession and employer-focused planning. Currently, the development of a national HHR database is in progress, and identifying the critical data necessary to understand the entire pharmacy workforce and provide evidence for future planning is a priority. Without such a centralized and national database, provinces, regions, systems and employers will be forced to plan in isolation and without coordination, leading to misallocation of human resources.
5. *Productivity and models of care:* While the importance of data-driven, evidence-based HHR planning is clear, there is also an additional layer of relevance: a vision for a Canadian health care system grounded on principles such as accessibility, equity, teamwork, patient-centredness and optimizing scopes and roles. Pharmacy advocates argue that this is the area of greatest immediate importance: a strong voice for pharmacy at decision- and policy-making tables that speaks with authority, backed by evidence and on behalf of the entire profession is essential. Significant advances have been made in this area, including greater coordination of national and provincial advocacy groups, engagement of large corporate employers in HHR activities, integration of regulatory bodies in workplace, workflow, workforce planning and so forth. Compared with other large health professions, pharmacy possesses some unique advantages in this area, including a relatively high degree of cooperation among different arms of the profession, the existence of large pan-Canadian umbrella organizations (including the Canadian Pharmacists’ Association, the National Association of Pharmacy Regulatory Authorities and the Association of Faculties of Pharmacy of Canada) that work to develop consensus within and across organizations and a track record of cooperation and adaptability in both unionized- and nonunionized workplace settings.

## The importance of HHR planning for individual pharmacists and employers

HHR planning may seem like a bureaucratic impossibility or an activity beyond the range of an “average” pharmacist. The recognition that good, and bad, HHR planning has a direct impact on the quality of every pharmacist’s working life and the care that gets provided to every patient in the country is essential. Whether individual pharmacists will choose to learn more about HHR planning and engage more fully in the process is difficult to predict. When we consider the litany of issues and complaints facing the profession, it is sobering to recognize the common thread of HHR planning. For example, the seemingly never-ending “boom-and-bust” cycle of shortages and surpluses of pharmacists (which leads to significant fluctuation in salaries and benefits), or the ongoing issues faced by international pharmacy graduates attempting to become licensed in Canada, or concerns about additional scope-of-practice workload and responsibilities without supports, resources or remuneration are all dimensions of HHR planning. Determining the role and optimal scope of regulated pharmacy technicians, nonregulated pharmacy assistants and the contributions of pharmacy learners during advanced pharmacy practice experience rotations are also underappreciated aspects of HHR planning.

Awareness of the 5 core areas of focus defined by Health Canada and recognizing their connection to a robust national HHR data set focused on the pharmacy professions highlights the important work ahead. Each individual pharmacist—in practice or outside of it—represents many different data points within this data set. These points and this set will ultimately be used to generate policies and drive decisions that in turn will shape the day-to-day work of pharmacists, the education they receive, the licensure requirements under which they practise and the workflow and design of workplaces.

## Summary

There is currently significant interest in HHR planning in pharmacy. Historically, HHR planning data in pharmacy have been underdeveloped and highly siloed. While individual employers and some provinces maintained some data sets relevant to HHR and planning, these have been uncoordinated, not integrated and reflected local rather than national health system interests and needs. As pharmacy has become more integral to primary care and other health systems and as the scope of practice responsibilities for pharmacists have increased in response to HHR crises, there is an urgent need for the pharmacy professions to integrate and align with HHR data sets in medicine, nursing and other professions more fully. This will be challenging and require overcoming inertia, historical practices and competitive intraprofessional interests. Shifting the profession’s focus away from single practices and organizations and even regions and provinces toward a more national perspective will allow pharmacy to engage with other professions more fully and HHR planning in a coordinated and national manner, to address the problems and questions that affect all pharmacists today and the care and service they provide in their communities.

The next article in this series will focus on the minimum data sets needed and used by Health Canada to inform HHR planning processes. Understanding what data are needed, how they are captured and interpreted and how they are applied to policy formulation and decision-making can help the profession more effectively mobilize and leverage its resources to support proper planning for the pharmacy professions in the future. ■
